# Supplementary figures and images for: Eye lens crystallin proteins inhibit the autocatalytic amyloid amplification nature of mature α-synuclein fibrils
Source: PLoS One. 2020 Jun 29;15(6):e0235198. doi: 10.1371/journal.pone.0235198 (PMC7323979; doi:10.1371/journal.pone.0235198)

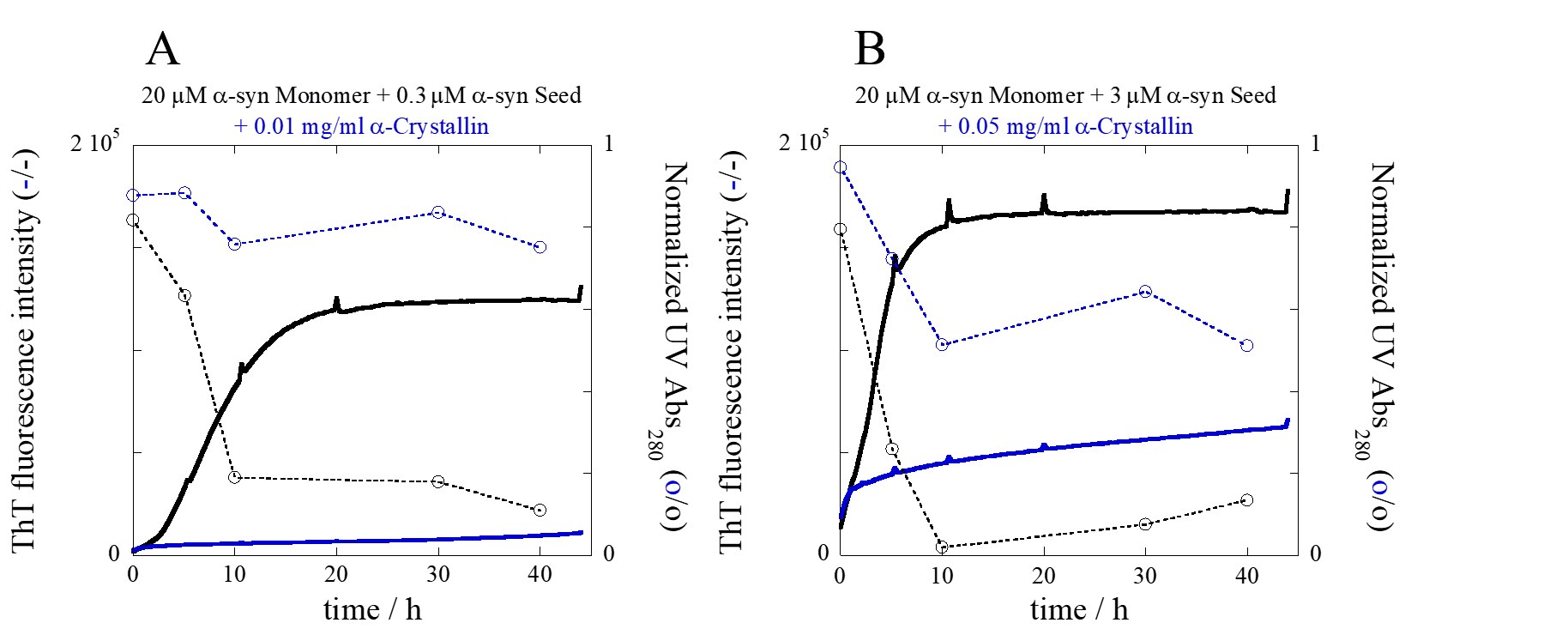

Supplement: S1 Fig — The seeded aggregation kinetics for 20 μM α-syn alone (black lines) and in the presence of α-crystallin (blue lines) was monitored using ThT. In parallel, samples along the aggregation reaction were aliquoted and centrifuged for 10 minutes at 13,000 g. The monomer concentration in the supernatant was measured by UV absorbance at 280 nm (open symbols and dashed lines). (A) 0.3 μM α-syn seeds alone and in the presence of 0.01 mg/ml α-crystallin; (B) 3 μM α-syn seeds alone and in the presence of 0.05 mg/ml α-crystallin. The experimental averages of at least three experimental repeats are shown. Important to highlight, the small artifacts seen in the ThT traces are due to the opening of the plate reader to remove samples for monomer concentration determination. (TIF) [file pone.0235198.s001.tif]

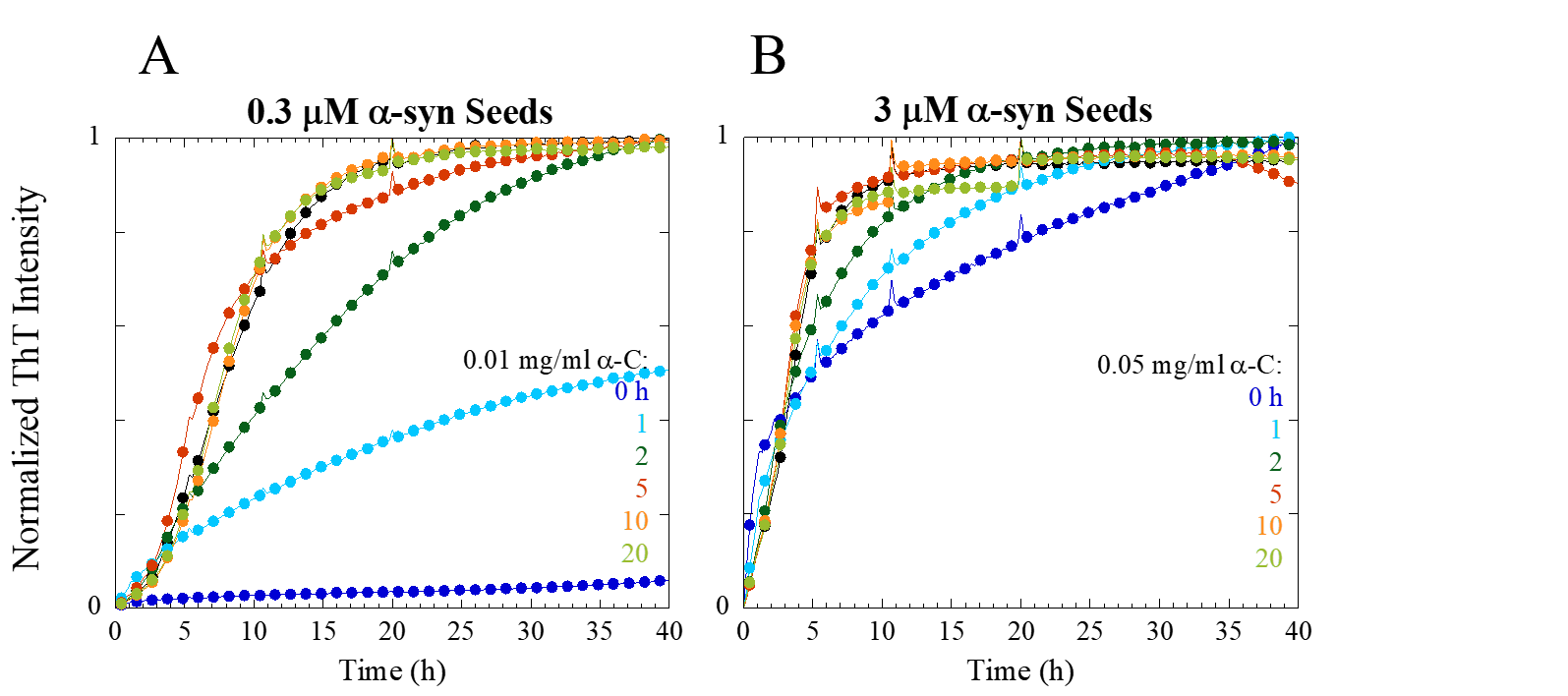

Supplement: S2 Fig — Seeded aggregation kinetics was monitored using ThT for 20 μM α-syn alone (black traces) and in the presence of two different α-crystallin concentrations (A) 0.01 mg/ml and (B) 0.05 mg/ml in 10 mM MES buffer pH 5.5 under quiescent conditions at 37°C. α-Crystallin was added at different time points along the aggregation reaction, 0, 1, 2, 5, 10 and 20 h. The average of at least three experimental repeats is shown. (TIF) [file pone.0235198.s002.tif]

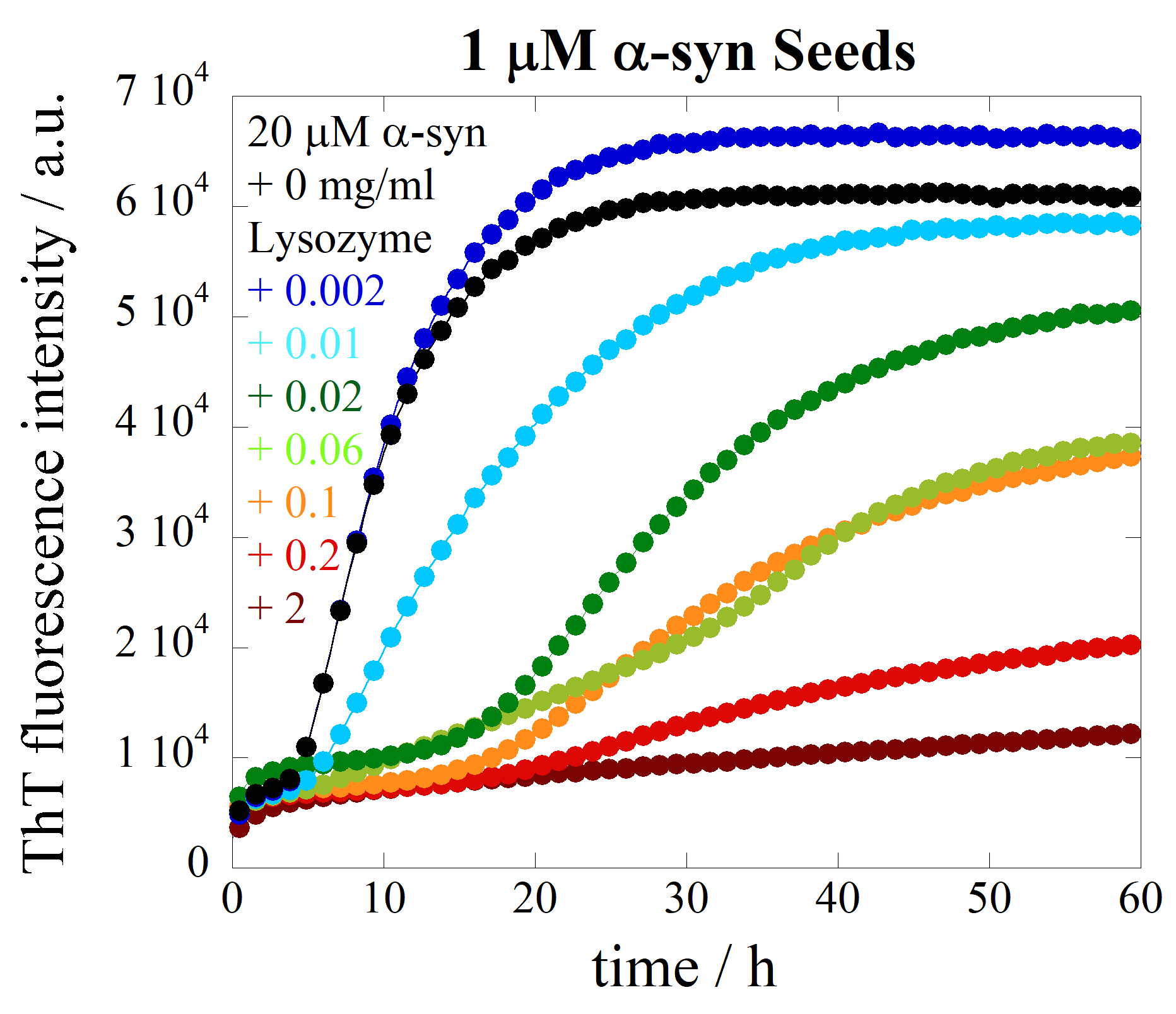

Supplement: S3 Fig — The aggregation kinetics of 20 μM α-syn monomer was monitored using ThT fluorescence in the presence of 1 μM seed concentration with Lysozyme concentrations ranging from 0.002 to 2 mg/ml in 10 mM MES buffer pH 5.5 under quiescent conditions at 37°C with color codes shown to the left. The figures show the average trace of at least three experimental repeats. (TIF) [file pone.0235198.s003.TIF]

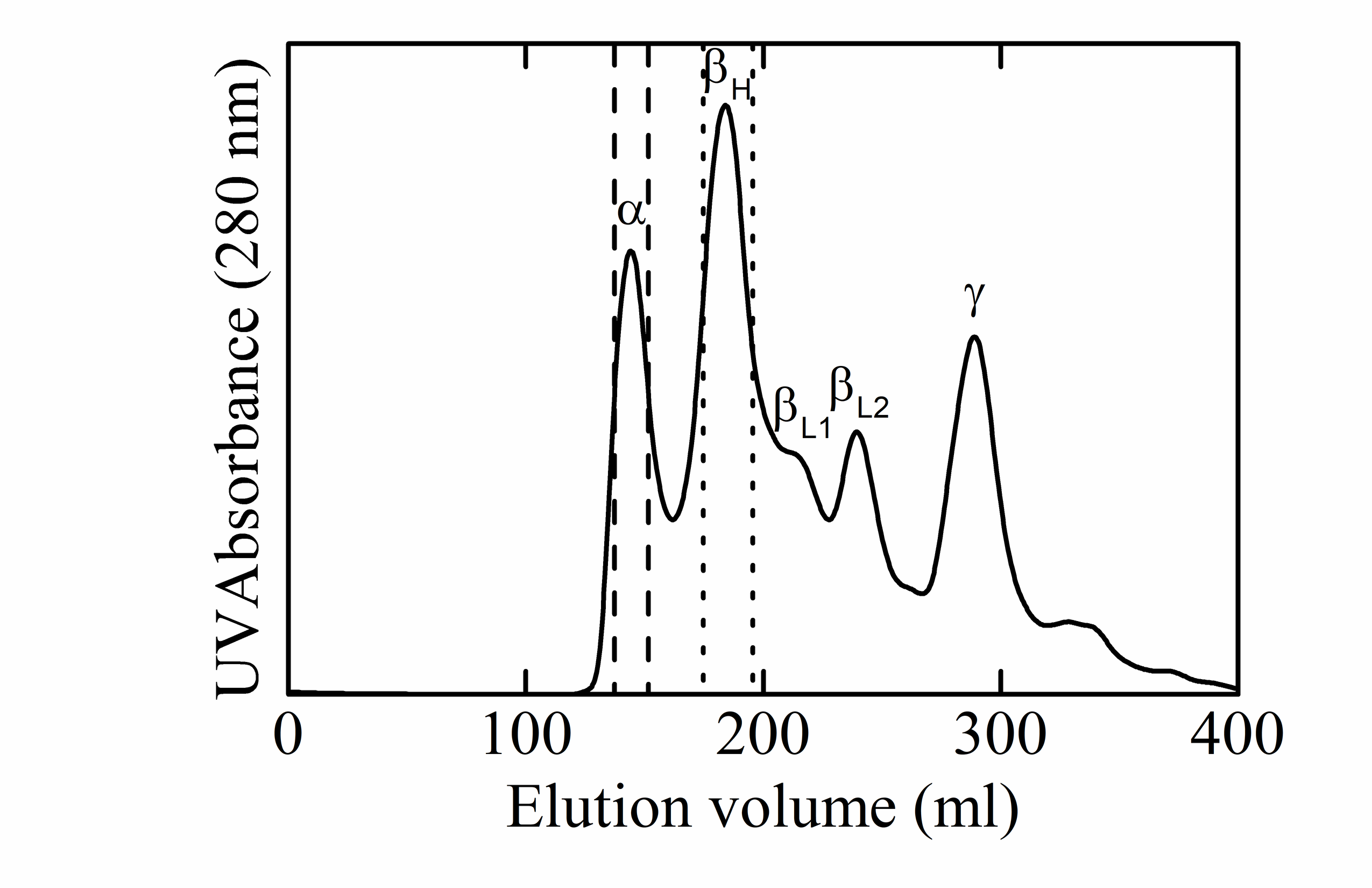

Supplement: S4 Fig — Chromatogram of cortical calf eye lens extract after passing through the SEC column which allows for the extraction of α- and βH-crystallin, with the collected fractions indicated by the vertical lines. Also visible are low molecular weight β variants, as well as a mixture of γ-crystallin variants. (TIF) [file pone.0235198.s004.tif]

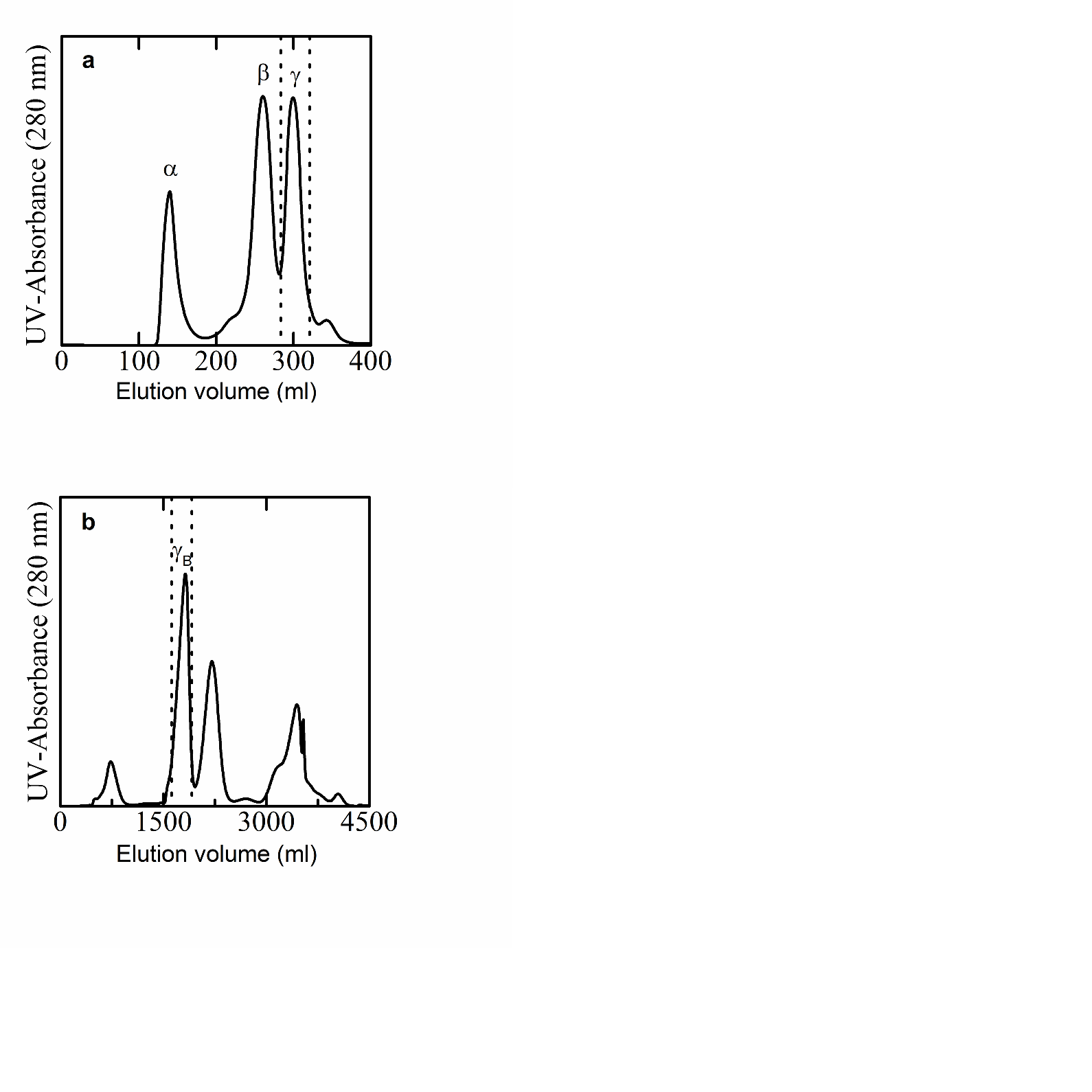

Supplement: S5 Fig — (a) A typical chromatogram of the nuclear calf eye lens extract after having passed a SEC column where a mixture of γ-crystallin variants is obtained by collecting the indicated elution volume. (b) Resulting chromatogram from an IEX column after passing the mixture of γ-crystallin variants shown in (a). This allows for the collection of purified γB-crystallin while the other variants of γ-crystallin are discarded. (TIF) [file pone.0235198.s005.tif]

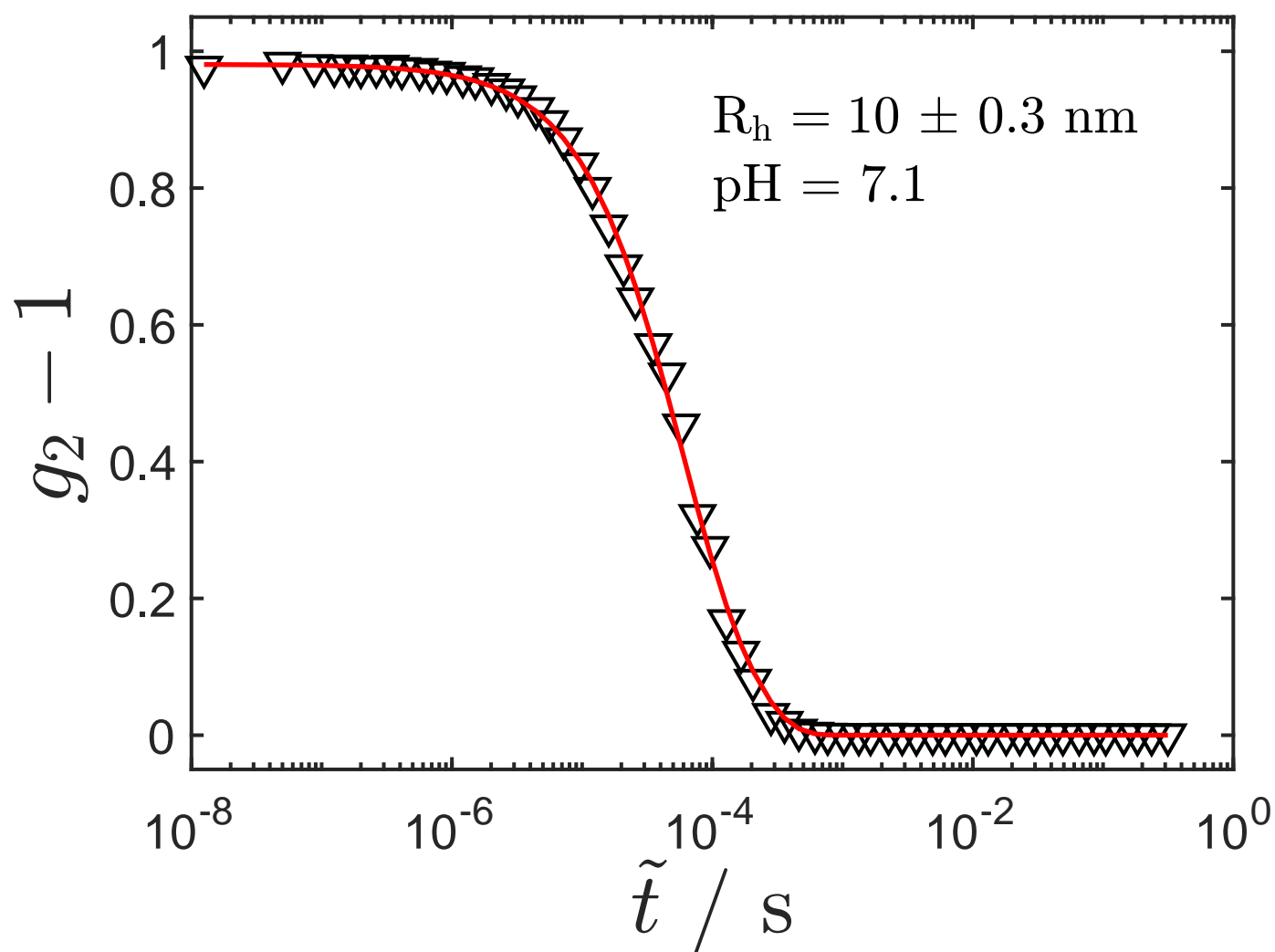

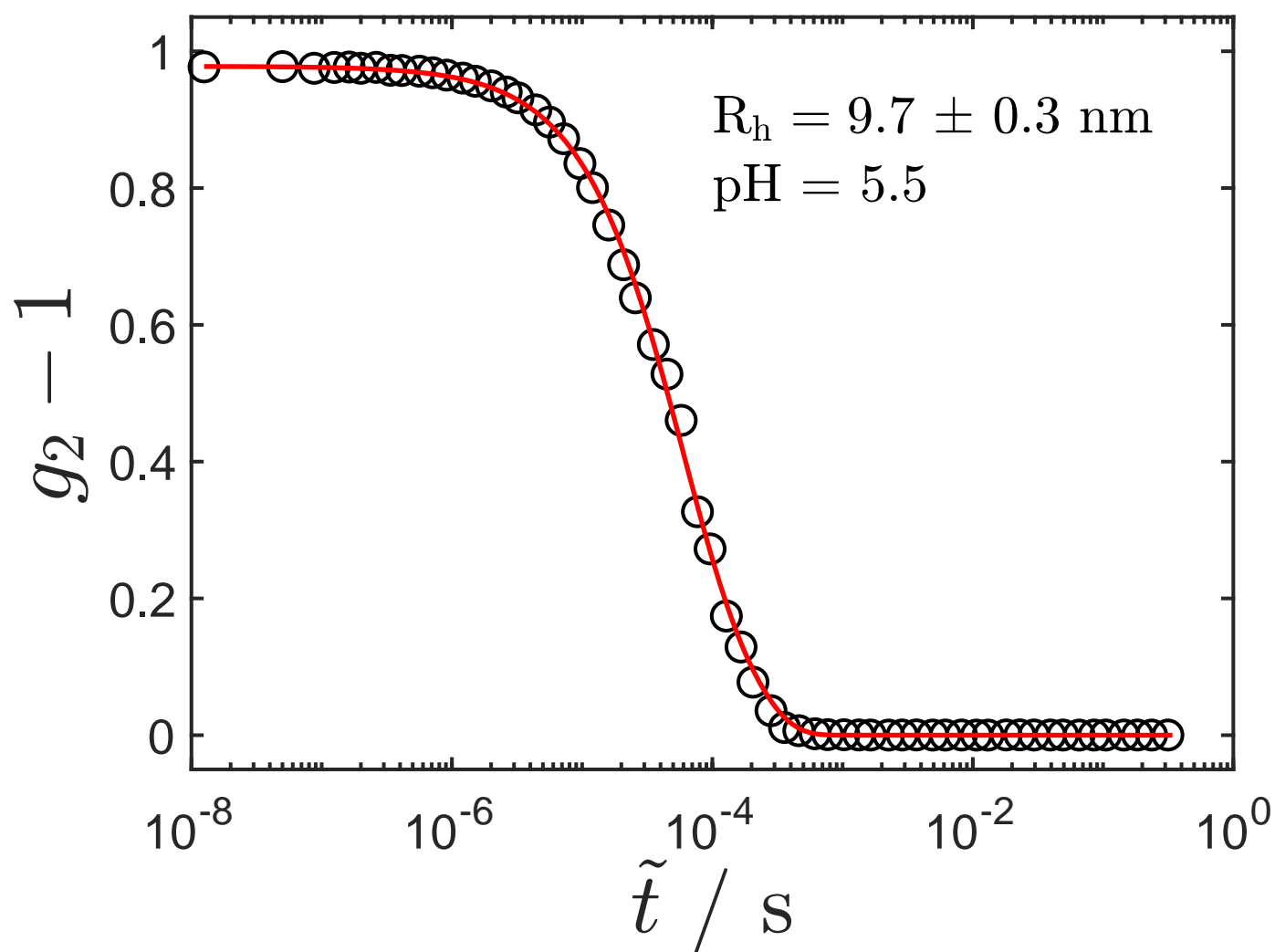

Supplement: S6 Fig — Correlation functions from DLS measurements on α-crystallin solutions at pH 7.1 (left) and pH 5.5 (right). Open symbols: data; red line: 2nd order cumulant fit. See text for details (S3 Methods in S1 Text). (PDF) [file pone.0235198.s006.pdf]

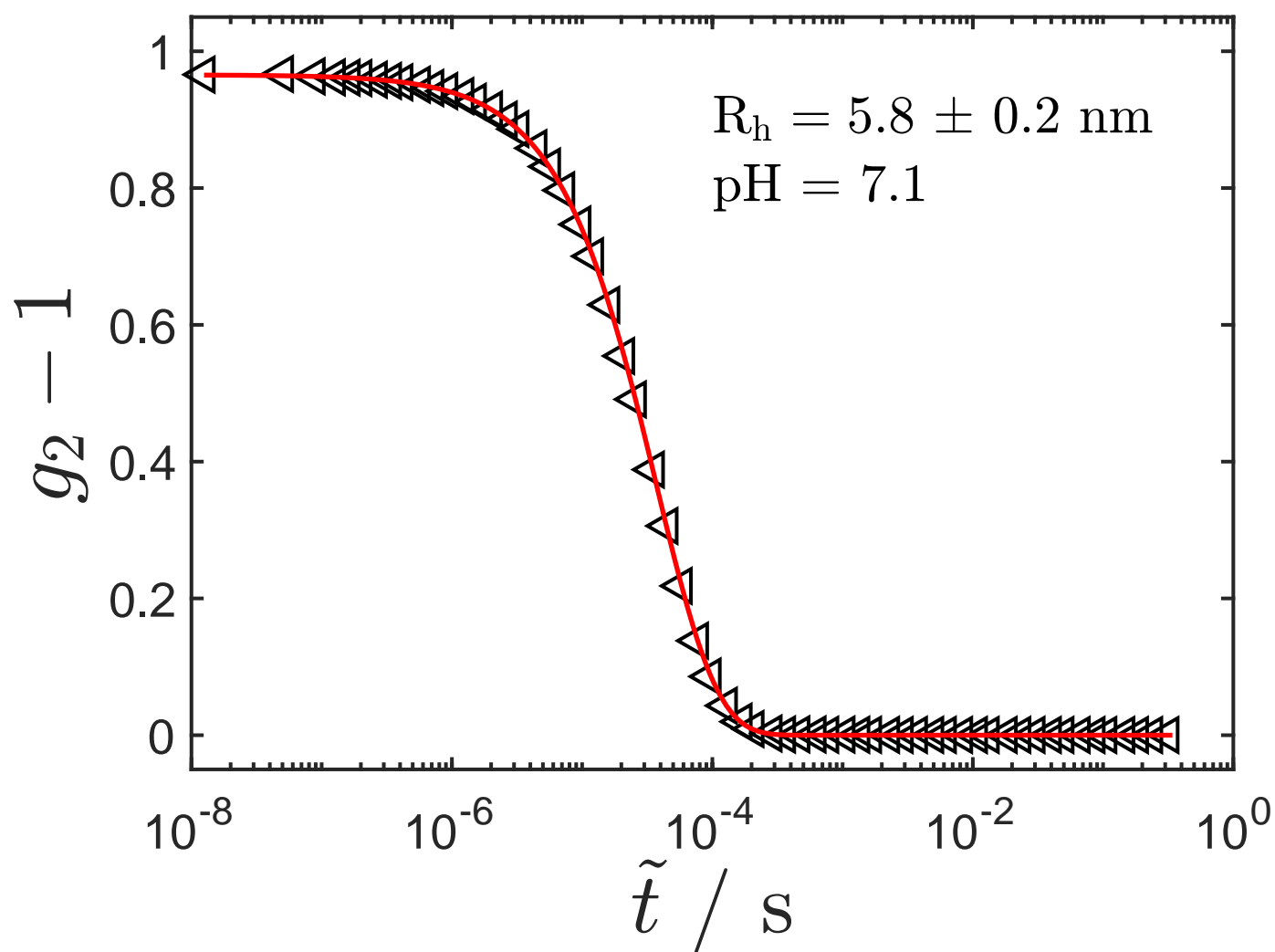

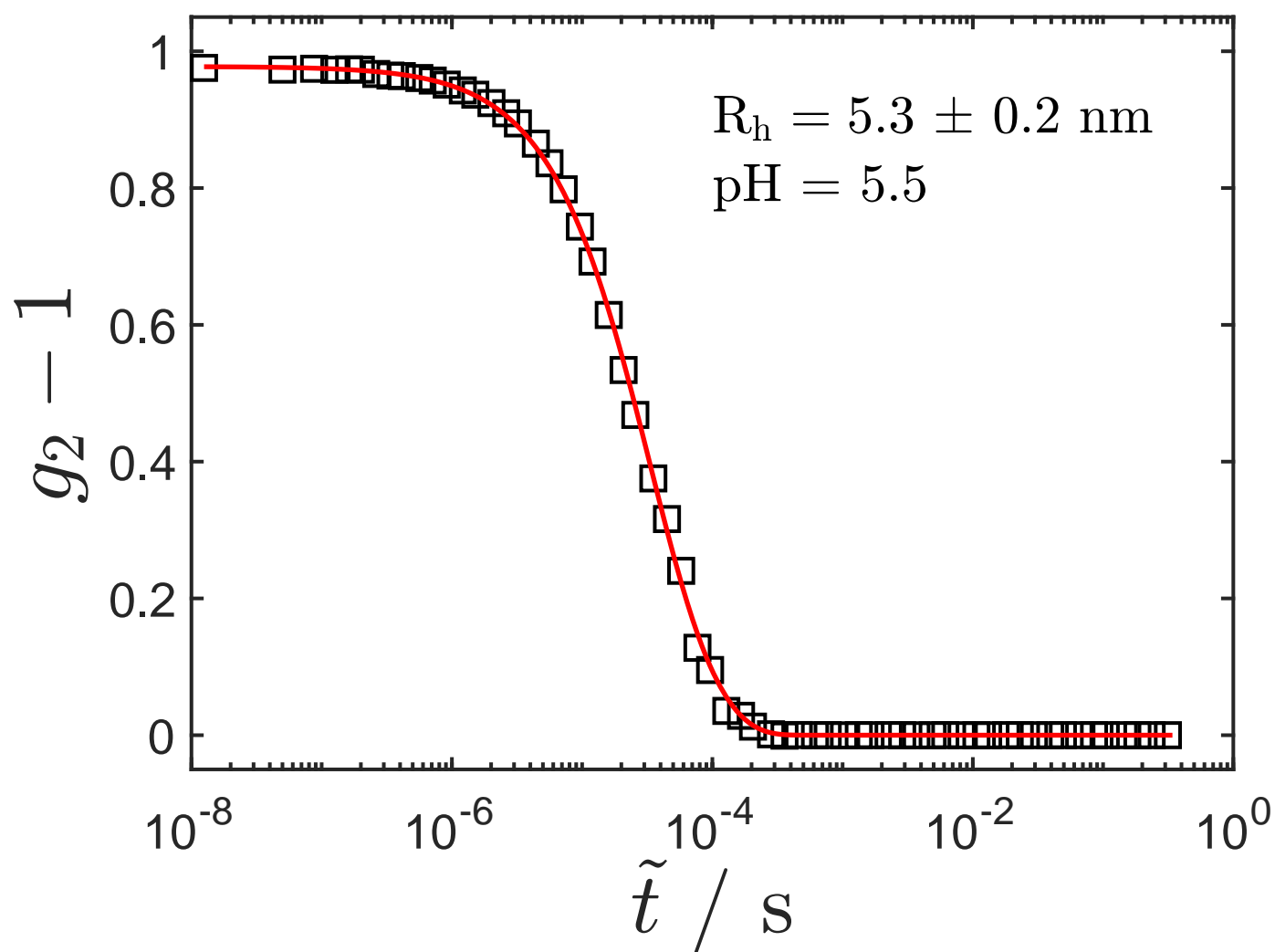

Supplement: S7 Fig — Correlation functions from DLS measurements on βH-crystallin solutions at pH 7.1 (left), and pH 5.5 (right). Open symbols: data; red line: 2nd order cumulant fit. See text for details. (PDF) [file pone.0235198.s007.pdf]
