# Supplementary material for: Eye lens crystallin proteins inhibit the autocatalytic amyloid amplification nature of mature α-synuclein fibrils
Source: PLoS One. 2020 Jun 29;15(6):e0235198. doi: 10.1371/journal.pone.0235198 (PMC7323979; doi:10.1371/journal.pone.0235198)
Supplement: S1 Text — (DOCX) [file pone.0235198.s008.docx]

**S1 Methods. α-Synuclein Solubility Measurements.** Samples taken along the α-synuclein (α-syn) aggregation process were aliquoted and centrifuged (10 min, 13000g). The α-syn protein concentration in solution for each supernatant was measured by UV absorbance at 280 nm. At least three measurements per sample were performed using the Thermo Scientific Nanodrop 2000 spectrophotometer. Time points from the lagphase, exponential growth phase and plateau were pin-pointed by following in parallel well controlled ThT aggregation kinetics.

**S2 Methods. Overall procedure for the purification of Crystallin proteins.** Fresh calf eyes are obtained from a slaughterhouse in Switzerland and the lenses are subsequently surgically removed and rinsed in MiliQ-H_2_O. The hard lens nucleus is separated from the softer cortical surroundings as their protein composition are differently suitable for the three types of crystallins. The cortical is stored at 4ºC in a phosphate buffer, 52.4 mM at pH 7.1 (18.4 mM NaH_2_PO_4_; 34 mM Na_2_HPO_4_; 50 mM NaCl; 1 mM DTT; 1 mM EDTA; 0.02 wt% NaN_3_) while the nuclear is stored at 4ºC in an acetate buffer at pH 4.5 (275 mM CH_3_COOH; 100 mM NaOH; 0.02 wt% NaN_3_). These buffers are also used as elution buffers in the subsequent SEC purifications. The two different batches are separately grinded to break down the cell walls and release the proteins, with chilled centrifugation being used to separate the protein extract from the biological residues. A HiLoad XK 26/60 Superdex 200 prep grade SEC column by GE Healthcare is used for both of the extracts, which have been freshly filtered before loaded onto the column and the concentration adjusted to suit the chromatography setup. The resulting chromatograms can be seen in figures S4 and S5, respectively which details the various components, as well as, the approximate volume fractions that would be retrieved and subsequently stored at 4ºC. The component retrieved from the nuclear extract with SEC consists of a mixture of different γ crystallins and is further separated using an IEX column (S5), GE Healthcare SP Sepharose fast flow ion exchange column. The fractions corresponding to γ_B_ are retrieved with the buffer afterwards replaced with phosphate and stored at 4ºC. Buffer exchange was performed using Amicon Ultra Centrifugal filters with a cut-off at either 10 kDa or 3 kDa depending on protein size. These centrifugal filters were also used to adjust the concentration when needed with the concentration being determined using UV absorption spectroscopy.

**S3 Methods. Hydrodynamic radius R_h_ of crystallin proteins at pH=7.1 and 5.5**. The hydrodynamic radius R_h_ of the multisubunit α-crystallin in dilute solution as obtained from Dynamic Light Scattering (DLS) experiments remains almost constant within the error bar when changing the pH from 7.1 (phosphate buffer used during the SEC preparation procedure) to 5.5 (MES buffer used for the aggregation kinetics experiments). A cumulant analysis of the correlation functions shown in Fig S6 leads to values for R_h_ of ca. 9 - 10 nm (pH 7.1: R_h_ = 10 ± 0.3 nm; pH 5.5: R_h_ = 9.7 ± 0.3 nm) with a polydispersity of 30 - 40% in good agreement with literature [1]. In the case of the multisubunit β_H_-crystallin proteins we observe a very slight decrease of the hydrodynamic radius from R_h_ = 5.8 ± 0.2 nm at pH = 7.1 to R_h_ = 5.3 ± 0.2 nm at pH = 5.5 as obtained from a cumulant analysis of the DLS correlation functions shown in Fig S7. The polydispersity of β_H_-crystallin is ca. 40 - 50% at both pH values. Changing the pH of solutions of the compact monomeric and thus perfectly monodisperse γB-crystallin proteins from neutral to 5.5 has no effect on the hydrodynamic radius (R_h_ = 2.3 nm as obtained from Dynamic Light Scattering experiments on the γB-crystallin batch used in this work and in agreement with previous batches as reported in literature [2] but only on the charge state and thus on the interprotein interaction potential and the protein phase behaviour [3]. DLS experiments were performed on a 3D-LS setup by LS Instruments (Switzerland) at a scattering angle of 90°, a temperature of 25 °C and at crystallin concentrations of ca. 5 mg/mL.

**References SI**

1. Foffi G, Savin G, Bucciarelli S, Dorsaz N, Thurston GM, Stradner A et al. Hard sphere-like glass transition in eye lens α-crystallin solutions. *PNAS* 2014; 111:16748–16753.

2. Bucciarelli S, Casal-Dujat L, De Michele C, Sciortino F, Dhont J, Bergenholtz J, Farago B, Schurtenberger P and Stradner A. Unusual Dynamics of Concentration Fluctuations in Solutions of Weakly Attractive Globular Proteins. *Journal of Physical Chemistry Letters* 2015; 6:4470–4474.

3. Bonnete F, Malfois M, Finet S and Tardieu A. Different Tools to Study Interaction Potentials in γ-Crystallin Solutions: Relevance to Crystal Growth. *Acta Cryst D* 1997; 53:438.
